# Treatment of the First Acute Relapse Following Therapeutic Plasma Exchange in Formerly Glucocorticosteroid-Unresponsive Multiple Sclerosis Patients—A Multicenter Study to Evaluate Glucocorticosteroid Responsiveness

**DOI:** 10.3390/ijms18081749

**Published:** 2017-08-11

**Authors:** Johannes Ehler, Stephan Blechinger, Paulus S. Rommer, Sebastian Koball, Steffen Mitzner, Hans-Peter Hartung, Fritz Leutmezer, Martin Sauer, Uwe K. Zettl

**Affiliations:** 1 Department of Anaesthesiology and Intensive Care Medicine, University of Rostock, 18057 Rostock, Germany; martin.sauer@uni-rostock.de; 2 Department of Neurology, Neuroimmunology Section, University of Rostock, 18147 Rostock, Germany; uwe.zettl@med.uni-rostock.de; 3 Department of Neurology, Medical University of Vienna, 1090 Vienna, Austria; stephan.blechinger@meduniwien.ac.at (S.B.); paulus.rommer@meduniwien.ac.at (P.S.R.); fritz.leutmezer@meduniwien.ac.at (F.L.); 4 Division of Nephrology, Department of Internal Medicine, University of Rostock, 18057 Rostock, Germany; sebastian.koball@uni-rostock.de (S.K.); steffen.mitzner@med.uni-rostock.de (S.M.); 5 Department of Neurology, Medical Faculty, Heinrich-Heine-University, 40225 Düsseldorf, Germany; hans-peter.hartung@uni-duesseldorf.de

**Keywords:** steroids, treatment response, demyelination, disease modifying therapies, outcome measurement

## Abstract

Therapeutic options to treat multiple sclerosis (MS) relapses comprise glucocorticosteroids (GCS) as first-line and therapeutic plasma exchange (TPE) as second-line treatments in GCS-unresponsive patients. No guidelines exist for the treatment of another relapse following TPE. We retrospectively analyzed the responsiveness to GCS in a subsequent relapse following TPE in previously GCS-unresponsive MS patients. Thirty-seven patients with GCS-unresponsive MS relapses received TPE (relapse A). All patients developed another relapse after the completion of TPE and received GCS again (relapse B). The primary study endpoint was the clinical response to GCS and TPE. Marked improvement was defined as clinically significant improvement in function, moderate improvement as a definite change of symptoms without significant impact on function, no effect comprised unchanged symptoms, and deterioration a worsening of symptoms or new deficits. The secondary endpoint was an improvement in expanded disability status scale (EDSS) scoring. All patients were GCS-unresponsive during relapse A and received TPE. During GCS treatment of relapse B, marked improvement was observed in 10, moderate improvement in 24, and no effect in three patients. The EDSS decreased in 15 patients. GCS might remain the first-line relapse treatment following TPE in formerly GCS-unresponsive MS patients.

## 1. Introduction

Multiple sclerosis (MS) is a chronic demyelinating disease of the central nervous system (CNS) [1,2]. The presence of more than 2.5 million MS patients worldwide and over 130,000 patients in Germany reflect the high socioeconomic importance of the disease [1]. Despite encouraging advances in drug treatment, MS remains a leading cause of disability and early retirement among young adults [3,4]. Both the prevention of disease progression and the treatment of severe neurologic impairment in MS patients are a challenge for physicians [1,5,6]. During the past 20 years, most research has focused on expanding the number of disease-modifying drugs (DMD). Current treatment options include interferons, glatiramer acetate, dimethyl fumarate, teriflunomide, fingolimod, and different monoclonal antibodies such as natalizumab, alemtuzumab, and daclizumab [5,7,8]. Despite the benefits of DMD, significant advances in the management of acute MS relapses have not been achieved. Alternatives are limited and include oral or intravenous administration of high-dose glucocorticosteroids (GCS) as a first-line treatment [7,9,10,11]. Therapeutic plasma exchange (TPE) is a second-line treatment for GCS-unresponsive relapses [7,10]. GCS potently suppress neuroinflammation and have become the standard treatment for acute relapses of MS and initial clinical attacks suggestive of MS [9,12,13]. The mechanisms of action within the immune system are pleiotropic, but a major impact appears to result from GCS-induced apoptosis of peripheral blood leucocytes, which might terminate neuroinflammation through the downregulation of T-cell activity [13]. Characteristically, the treatment effects of GCS are delayed, with a peak effect within 7–10 days after a 5-day course of administration. Therefore, the response to GCS should be evaluated at least two weeks after the initiation of relapse treatment [10]. In GCS-unresponsive patients, TPE can be performed [7,10]. The rationale for this extracorporeal blood purification technique is the removal of circulating antibodies, cytokines, immune complexes, and complementary factors, all of which are assumed to be involved in immune-mediated neuroinflammation [9,14,15,16,17]. In contrast to GCS-induced T-cell apoptosis, TPE also exerts major beneficial effects on disturbed humoral immunity [14]. The aim of the current study was to evaluate GCS-responsiveness before and after TPE to determine whether formerly GCS-unresponsive patients are GCS-responsive again after TPE, or whether they remain GCS-unresponsive during a subsequent relapse.

## 2. Results

### 2.1. Baseline Characteristics and Magnetic Resonance Imaging Findings before the Initiation of Treatment for Relapse A

Between 2001 and 2014, a total of 37 patients with GCS-unresponsive MS relapses were treated with TPE. The population consisted of 29 female and eight male patients. Six patients with clinically isolated syndrome (CIS), 24 with relapsing-remitting MS (RR-MS), six secondary-progressive MS (SP-MS) patients with superimposed relapses, and one primary-progressive MS (PP-MS) patient with acute worsening symptoms were included. DMD were used by 20 of the 37 patients (54.1%). The DMD included interferon beta 1-a in six patients, intravenous immunoglobulin in three patients, mitoxantrone in three patients, azathioprine in one patient, natalizumab in two patients, intermittent GCS treatment (1 g methylprednisolone (MP)/day over 5 days every three months) in one patient, rituximab in one patient, cyclophosphamide in one patient, and fingolimod in two patients. At the time of relapse A, a single functional system (monosymptomatic relapse) was affected in five of the 37 patients (13.5%). In 32 of the 37 patients (86.5%), multiple functional systems (polysymptomatic relapse) were affected. In 31 of the 37 patients (83.8%), complete magnetic resonance imaging (MRI) data (MRI reports from experienced neuro radiologists) acquired before the initiation of treatment of relapse A were available. An overview of the baseline characteristics and MRI findings is shown in Table 1.

### 2.2. GCS Treatment for Relapse A

**Figure 1:**
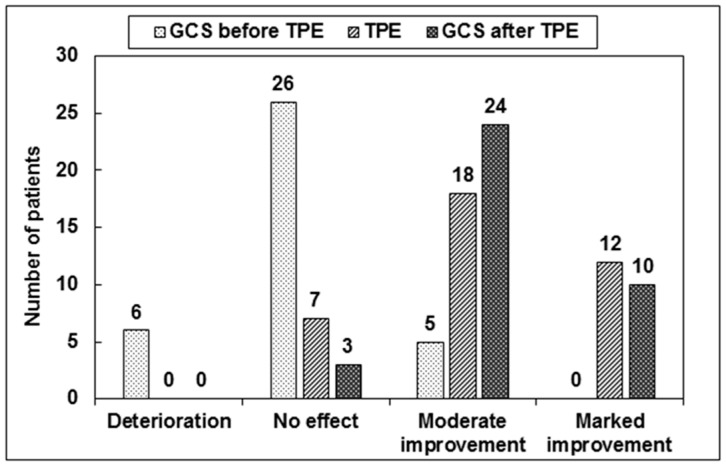
Different response to glucocorticosteroid (GCS) treatment before and after therapeutic plasma exchange (TPE) in 37 clinically isolated syndrome (CIS) and multiple sclerosis (MS) patients. Deterioration was defined as worsened target neurologic deficit or new neurologic symptoms, marked improvement as clinically significant improvement in function, moderate improvement as a definite change of the neurologic deficit without significant impact on function within the functional score, and no effect as unchanged symptoms.

All 37 patients received a conventional high-dose GCS treatment (1 g MP/day over 5 consecutive days) for relapse treatment. The median time from relapse onset to the start of GCS treatment was 7 days (range 1–60 days). An “ultra” high-dose GCS treatment (2 g MP/day given daily over 5 consecutive days) was administered additonally to 18 of the 37 patients (48.7%). For 19 of the 37 patients, TPE was performed without an “ultra” high-dose GCS treatment due to rapid disease progression, severe adverse events due to GCS, prior GCS treatment in an external hospital, or unresponsiveness to GCS during intermittent GCS treatment. The median cumulative GCS dose was 10 g MP/patient (range 5–15 g). The affected functional systems included visual function in four patients, brainstem function in four patients, sensory function in three patients, spinal cord sensorimotor function in nine patients, cerebellar function in six patients, pyramidal function in 10 patients, and bowel and bladder function in one patient. Regarding our primary endpoint of the response to GCS treatment, of a total of 37 patients, no patient achieved marked improvement, moderate improvement was observed in five, no effect was achieved in 26, and deterioration occurred in six patients. Based on our secondary endpoint, no patient achieved a response to GCS treatment. In six of the 37 patients (16.2%), adverse events occurred during the initial GCS treatment (osteoporosis with fracture in one, psychosis in one, gastrointestinal events in three, and dermatologic side effects (allergic exanthema) in one patient; subsequently, an equivalent dose of dexamethasone was utilized). The median expanded disability status scale (EDSS) score before and after GCS treatment for relapse A was 3.5 (range 2.0–7.5). The mean EDSS score was 4.4 (SD = 1.8) before and after GCS treatment for relapse A. The clinical response to GCS treatment for relapse A is shown in Figure 1 and Table 2.

### 2.3. TPE for Relapse A

A median of 5 single TPE sessions per patient was performed (range 2–9 single sessions). The median time from relapse onset to the initiation of TPE was 44 days (range 11–154 days). The median time between the end of GCS treatment and the beginning of TPE was 15 days (range 0–98 days). Based on our primary response definition, marked improvement was observed in 12 patients (32.4%), moderate improvement in 18 patients (48.7%) and no effect in seven of the 37 patients (18.9%) (Figure 1, Table 3). Regarding our secondary endpoint, a response to TPE was observed in 18 of the 37 patients (48.7%), and 19 patients were non-responsive to TPE (51.4%). The median number of single TPE sessions until a clinical response occurred was 5 (range 2–8 sessions). A comparison of median EDSS values before (EDSS score 3.5, range 2.0–7.5) and after TPE (EDSS score 3.5, range 1.0–7.0) showed a significant reduction in the EDSS score (*p* < 0.001). The mean EDSS value before TPE decreased from 4.4 (SD = 1.8) to 3.7 (SD = 1.9). Adverse events occurred in 10 of the 37 patients (27.0%), and are shown in Table 4. In all CIS patients a treatment with interferone-1b or glatiramer acetate was established following relapse A. DMD in patients with RR-MS and SP-MS were not changed following treatment of relapse A. Intermittent GCS treatment was continued in one PP-MS patient.

### 2.4. GCS Treatment for Relapse B

All 37 patients developed a new acute relapse after the completion of TPE. The median time from the beginning of relapse A to the beginning of relapse B was 150 days (range 31–2588 days), while the median time was 90 days (range 4–2487 days) from their final TPE session to relapse B. At the time of relapse B, the study population consisted of 29 RR-MS patients, seven SP-MS patients, and one patient with PP-MS (six former CIS patients were attributed to the RR-MS patients group, one former RR-MS patient underwent a transition to SP-MS). The affected functional systems included visual function in four patients, brainstem function in four patients, sensory function in six patients, spinal cord sensorimotor function in three patients, cerebellar function in three patients, and pyramidal function in 17 patients. A monosymptomatic relapse was documented in 13 (35.1%) and a polysymptomatic relapse occurred in 24 of the 37 patients (64.9%). The median duration from relapse onset until the start of GCS treatment was 6 days (range 0–122 days).

Thirty-five of the 37 patients (94.6%) received high-dose GCS treatment with 1 g MP/day over 5 consecutive days. Two patients (5.4%) with a new severe relapse immediately received 2 g MP/day over 5 consecutive days. In eight of the 37 patients (21.6%), an ultra-high-dose GCS treatment was administered after the completion of the initial high-dose GCS treatment with 1 g MP due to persistent neurological deficits. The median cumulative GCS dosage was 5 g MP/patient (range 5–15 g). Based on our primary endpoint, a marked improvement was achieved in 10 (27.0%) patients, a moderate improvement occurred in 24 (64.9%) patients, and no effect was observed in three of the 37 (8.1%) patients (Figure 1). Regarding the secondary endpoint, a GCS response was achieved in 15 of the 37 (40.6%) patients. The median EDSS values before (EDSS 4.5, range 2.0–8.0) and after GCS treatment for relapse B (EDSS 3.5, range 1.5–7.5) showed a significant EDSS reduction (*p* < 0.001). The mean EDSS values decreased from 4.6 (SD = 1.7) before to 4.0 (SD = 1.8) after GCS treatment. An overview of the response to GCS treatments and TPE is shown in Table 2, Table 3 and Table 5. Adverse events occurred during GCS treatment for relapse B in two of the 37 patients (5.4%, one gastrointestinal and one dermatologic (allergic exanthema) event).

**Figure 2:**
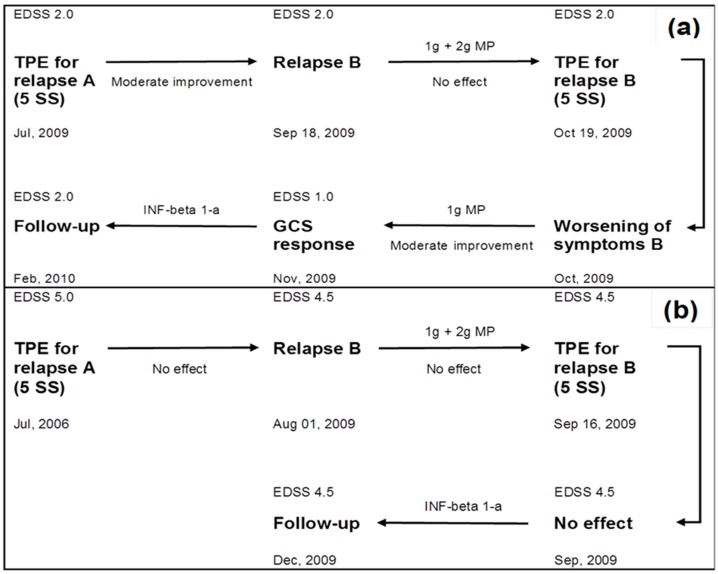
Extended treatment with glucocorticosteroids (GCS) and repeated therapeutic plasma exchange (TPE) in two GCS-unresponsive multiple sclerosis (MS) patients. (**a**) patient one with relapsing-remitting (RR)-MS; (**b**) patient two with a transition from clinically isolated syndrome (CIS) to RR-MS between relapse A and relapse B. Moderate improvement was defined as a definite change of the neurologic deficit without significant impact on function within the functional score, and no effect as unchanged symptoms. 5 SS: Five single sessions.

Of three GCS-unresponsive patients during treatment of relapse B, two patients received a second TPE series (Figure 2a,b). The new relapse in patient one occurred 39 days after the completion of TPE for relapse A (Figure 2a). A GCS response for relapse B was achieved with another high-dose GCS treatment after the completion of the second TPE series. In patient two, relapse B occurred 1006 days after the completion of TPE (Figure 2b). In this patient, neither GCS treatment nor a second TPE series were able to improve the patient’s condition. In a third patient who underwent a transition from RR-MS to SP-MS, an intermittent GCS treatment (1 g MP/day over 5 days every three months) was established.

### 2.5. Follow-up Examination after GCS Treatment for Relapse B

**Figure 3:**
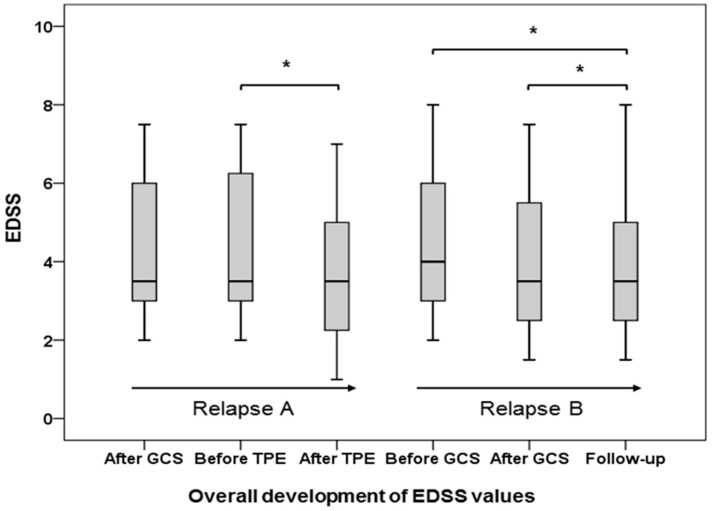
Boxplot graphs displaying the median (bold line), minimum (lower T-line), maximum (upper T-line), first quartile (lower part of box), and third quartile (upper part of box) of expanded disability status scale (EDSS) values during treatment of relapses A and B until follow-up examination. Relapse A: initial GCS-unresponsive relapse treated with GCS and TPE; Relapse B: first new relapse after TPE treated with GCS; * EDSS changes were significantly different (*p* < 0.001) in a non-parametric Wilcoxon test.

Thirty-six of the 37 patients (97.3%) were subjected to a clinical re-evaluation for GCS response after GCS treatment of relapse B. The median time to the follow-up examination was 72 days (range 9–209 days) after their final GCS treatment. The patients were re-evaluated for clinical symptoms (the target neurologic deficit, TND) and EDSS scores. The median EDSS score at the time of follow-up was 3.5 (range 1.5–8.0), which did not change significantly compared to EDSS values after the completion of GCS treatment for relapse B (median EDSS 3.5, range 1.5–7.5, *p* = 0.294). The mean EDSS value at follow-up (3.9, SD = 1.9) was slightly decreased compared to the EDSS value after GCS treatment of relapse B (4.0, SD = 1.8). The median EDSS values during all treatments are shown in Figure 3.

## 3. Discussion

We presented a series of 37 GCS-unresponsive patients who were clinically re-evaluated to determine GCS-responsiveness after the completion of TPE. Because clinicians have to choose among therapeutic options in the event of a subsequent relapse following TPE, the further development of GCS-responsiveness is of high interest. Reports of variable GCS-responsiveness in MS relapses before and after TPE are rare. Case reports have described clinical worsening during TPE in GCS-unresponsive MS relapses [18,19,20]. Previously, our group reported successful GCS treatment in formerly GCS-unresponsive MS and CIS patients with worsening symptoms during TPE who regained GCS responsiveness after TPE [18,21]. Based on the concept of a persistent, distinctive pattern of demyelination in MS lesions, a debate is ongoing regarding the variable GCS treatment responses within individual patients, as presented in our study [22]. The immunosuppressive effects of GCS are thought to be achieved through T-cell apoptosis [13]. Following the concept of an intra-individual homogeneity of MS patterns, the GCS effects should be comparable in the same patient, especially within short intervals of repeated treatment [22,23,24]. Keegan et al. observed a response to TPE exclusively in patients with pattern II lesions, which highlighted the importance of a distinctive pattern of demyelination in response to treatment in MS patients [16,23]. Our clinical observations of variable GCS treatment responses are not consistent with the concept of an intra-individual homogeneity of MS lesions [18]. This variable response to GCS in single patients does not support this neuropathologic division. Furthermore, Barnett et al. reported on newly forming lesions in patients with severe MS relapses and proposed that heterogeneity might be stage-dependent instead of patient-dependent [24,25]. Based on the present study, a stage-dependent lesion pathology with different responses to therapeutic interventions might be an explanatory hypothesis [18,26]. Additionally, treatment effects by GCS and TPE during relapse A with a possible re-establishment of nerve conduction in demyelinated nerves might be another explanation for the varying GCS responses observed during the treatment of relapse B. However, retrospective observational clinical studies are unable to answer these underlying immunopathological questions. From a purely neuropathological standpoint, only short-term sequential brain biopsies obtained from patients receiving GCS could reliably elucidate changes in active MS lesions and could explain the variability in GCS-responsiveness [23]. This is not justifiable from a clinical standpoint. Response to GCS treatment or TPE may also be influenced by other factors (e.g., sex or stage of disease), and will be greater at an earlier disease stage. Indeed, treatment response to GCS treatment or TPE was greater in CIS and RR-MS patients. However, whether GCS-unresponsive patients remain GCS-unresponsive during a subsequent relapse following TPE is highly important for clinicians. The high proportion of patients who regained GCS-responsiveness presented here might advocate for a primary re-treatment with GCS [10]. Despite the observation of only a small number of adverse events during TPE in the present study, extracorporeal treatment remains an invasive procedure with potentially severe complications [9,27,28]. Therefore, our data might be important because they favor GCS administration as a first-line treatment during a subsequent relapse after TPE.

In the present study, we focused primarily on the clinical changes in the TND and secondarily on EDSS changes, although quantitative EDSS rating is supposed to be favorable in comparison to subjective clinical examination. Pure evaluation of EDSS values, on the other hand, is limited by insufficient symptom representation in this score and can indicate response to GCS or TPE imprecisely [21]. We observed various patients with clinical improvement with regard to our primary response definition who had a corresponding decrease in the EDSS score that was certainly below the predefined “cut-off” in our secondary response definition. Therefore, these patients achieved clinical response to treatment but were non-responders regarding the EDSS response definition. The obvious differences between the two response definitions reveal that the accurate clinical response definition is better suited for the treatment with GCS and TPE. The unblinded clinical assessment of the response to treatment was a main limitation. The inconstant timing of treatment and of patient evaluation during follow-up represents a limitation of daily clinical practice. Despite these obvious shortcomings, our results impressively demonstrate GCS-responsiveness during relapse B, with marked improvement in 27% and at least moderate clinical improvement in another 65% of our patients. In comparison to 14% of patients with only moderate clinical improvement during the treatment of relapse A, this is highly interesting from a clinical view. Larger, prospective, multicenter trials of TPE in GCS-unresponsive patients are encouraged to investigate the potential predictors of regained GCS-responsiveness in MS relapses. Optimal treatment of relapses increases the chance of limiting or avoiding residual deficits which have been related to the progression of disability in MS [6]. Furthermore, clinical trials should investigate the potential direct impact of TPE on immune cells in regard to MS patients [14,29,30].

## 4. Materials and Methods

### 4.1. Study Participants and Inclusion Criteria

**Figure 4:**
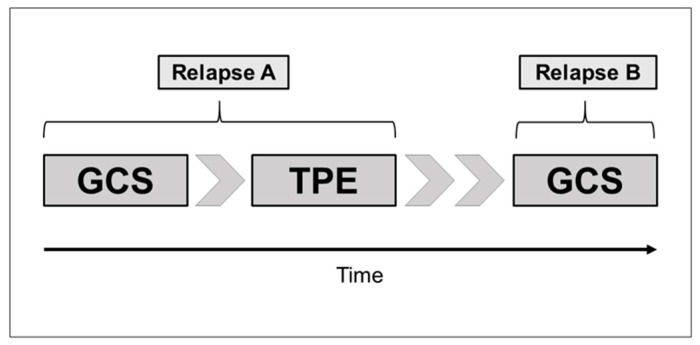
Treatment algorithm of 37 clinically isolated syndrome (CIS) and multiple sclerosis (MS) patients. Relapse A represented the initial, glucocorticosteroid (GCS)-unresponsive relapse treated with GCS and subsequent therapeutic plasma exchange (TPE); Relapse B represented the first acute relapse after TPE treated with GCS again.

Data regarding GCS and TPE treatment from 37 patients diagnosed with acute GCS-unresponsive MS relapses (2001 McDonald criteria) at two university hospitals with MS centers were retrospectively evaluated [13]. A new and definite clinical attack was defined as an acute relapse [31]. Pseudo-relapses were excluded from this investigation. GCS-unresponsiveness was defined as deteriorated, unchanged, or only moderately (insufficiently) improved symptoms following the completion of GCS treatment. The inclusion criteria consisted of unresponsiveness to GCS treatment before TPE (relapse A), subsequent TPE (relapse A), and GCS treatment for a subsequent relapse following TPE (relapse B), as shown in Figure 4. According to our relapse definition, seven SP-MS patients with superimposed relapses and one patient with PP-MS with acute worsening symptoms were evaluated. Twenty-four patients in the current analysis were part of a recently published analysis of 90 GCS-unresponsive TPE patients [32]. All patient data and recordings were anonymized and de-identified prior to our analysis. The study was conducted in accordance with the Declaration of Helsinki and approved by the local ethics board of Rostock University (identifier A 2015-0065, 29 May 2015) and by the Institutional Ethics Committee of the Medical University of Vienna (EK-NR 1660/2014, 2 September 2014).

### 4.2. Outcome Measures

The response to treatment was analyzed at least two weeks after the end of each patient’s final GCS treatment (relapse A and B) and each patient’s final TPE session.

#### 4.2.1. Primary Endpoint of the Response to GCS Treatment and TPE

The definition of the response to GCS treatment and TPE was primarily based on changes in the target neurologic deficit (TND, the predominant neurological symptom, assigned to a functional system) according to a clinical examination by an EDSS-certified neurologist [16,33]. Marked improvement was defined as a clinically significant improvement in function. Moderate improvement represented a definite change in the neurologic deficit without a significant impact on function. No effect indicated unchanged symptoms. Deterioration designated patients with worsened TND or new neurologic symptoms.

#### 4.2.2. Secondary Endpoint of the Response to GCS Treatment and TPE

The Expanded Disability Status Scale (EDSS) was used for clinical evaluation and scoring before, during, and after GCS treatment and TPE by EDSS-certified neurologists [34]. A response was defined as an EDSS score decrease ≥1.0 point in patients with an initial EDSS score ≤5.5 or an EDSS score decrease ≥0.5 points in patients with an initial EDSS score ≥6.0 [25].

### 4.3. Administration of GCS

Intravenous high-dose GCS were administered according to recommendations and involved a dosage of 1 g methylprednisolone (MP)/day over 5 consecutive days [10]. If definite improvement was not achieved within two weeks after the completion of high-dose GCS treatment, we considered the administration of a higher second intravenous GCS pulse with a dosage of 2 g MP/day over 5 consecutive days (ultra-high-dose GCS treatment) [10]. The response to ultra-high-dose GCS treatment was evaluated at least two weeks after completion of this treatment.

### 4.4. TPE Procedures

All 37 patients gave written informed consent for TPE and, if applicable, for central venous access (CVA) before the initiation of treatment. A minimum of three TPE sessions per patient were performed with an interval of two days between procedures. For severely affected patients, the first two TPE sessions were performed daily. Further treatments up to a maximum of nine TPE sessions were performed if no or only a moderate positive change in the patient’s TND was achieved. The plasma volume was estimated using nomograms [35]. A single plasma volume was exchanged at every treatment session (median 3.0 L, range 1.9–6.3 L). The TPE platforms in Rostock were as follows: Fresenius Multifiltrate, Fresenius Medical Care AG, Bad Homburg, Germany; Baxter BM 11/14, Baxter Healthcare, Deerfield, IL, USA; Gambro AB, Lund, Sweden; and Miltenyi Life18 system, Miltenyi Biotech, Bergisch-Gladbach, Germany. The TPE platform in Vienna consisted of the Spectra Optia Apheresis System (Terumo BCT, Lakewood, CO, USA).

### 4.5. Statistical Analysis

The comparison of continuous variables, including EDSS values before and after GCS (relapse A and B) and TPE (relapse A) treatment, was performed using non-parametric tests (Wilcoxon test). Statistical significance was indicated by *p* < 0.05 (two-sided) (IBM SPSS Statistics, Version 20, Chicago, IL, USA).

## 5. Conclusions

We here describe for the first time a series of GCS-unresponsive patients who regained GCS-responsiveness during a subsequent relapse following TPE. According to risk-benefit assessments, GCS might be more favorable than repeated TPE for subsequent relapses. Nevertheless, the pathophysiological mechanisms corresponding to the clinically observed heterogeneity of GCS-responsiveness remain to be elucidated.

## Figures and Tables

**Table 1:** Baseline characteristics of patients with clinically isolated syndrome (CIS) and Multiple sclerosis (MS) before treatment of relapse A.

Baseline Characteristics before Relapse A	CIS	RR-MS	SP-MS	PP-MS	All Patients
No. of patients	6	24	6	1	37
Age (years) ^a^	34.5 (20–46)	31.5 (15–52)	34.5 (25–69)	73.0	32.0 (15–73)
Disease duration (months) ^a^	1.0 (1–3)	63.0 (1–273)	101.0 (65–204)	291.0	65.0 (1–291)
Disease-Modifying Drugs (DMD) (%)	0.0	58.3	100.0	0	54.1
Relapses ≤ 12 months ^a^	1.0 (1)	2.0 (1–4)	1.0 (1–3)	1.0	2.0 (1–4)
T2-lesions in MRI (%) ^b^	6 (100)	21 (100)	3 (100)	1 (100)	31 (100)
Gd^+^ lesions in MRI (%) ^b^	6 (100)	12 (57)	2 (67)	0 (0)	20 (65)

^a^ Median (range); ^b^ Complete magnetic resonance imaging (MRI) data (T2-sequences and Gd-administration) available from 31 of 37 patients: six of six in CIS, 21 of 24 in relapsing-remitting MS (RR-MS), three of six in secondary-progressive MS (SP-MS) and one of one in primary-progressive MS (PP-MS).

**Table 2:** Clinical response to GCS treatment for relapse A in patients with CIS and MS.

MS Type at Relapse A	Marked Improvement (*n*)	Moderate Improvement (*n*)	No Effect (*n*)	Deterioration (*n*)
CIS (*n* = 6)	0	2	4	0
RR-MS (*n* = 24)	0	3	17	4
SP-MS (*n* = 6)	0	0	4	2
PP-MS (*n* = 1)	0	0	1	0
All patients (*n* = 37)	0	5	26	6

*n*: number; deterioration was defined as worsened target neurologic deficit or new neurologic symptoms; marked improvement as clinically significant improvement in function; moderate improvement as a definite change of the neurologic deficit without significant impact on function within the functional score; and no effect as unchanged symptoms.

**Table 3:** Clinical response to TPE for relapse A in patients with CIS and MS.

MS Type at Relapse A	Marked Improvement (*n*)	Moderate Improvement (*n*)	No Effect (*n*)	Deterioration (*n*)
CIS (*n* = 6)	3	2	1	0
RR-MS (*n* = 24)	9	11	4	0
SP-MS (*n* = 6)	0	4	2	0
PP-MS (*n* = 1)	0	1	0	0
All patients (*n* = 37)	12	18	7	0

*n*: number; deterioration was defined as worsened target neurologic deficit or new neurologic symptoms; marked improvement as clinically significant improvement in function; moderate improvement as a definite change of the neurologic deficit without significant impact on function within the functional score; and no effect as unchanged symptoms.

**Table 4:** Complications of TPE in GCS-unresponsive CIS and MS patients during treatment of relapse A.

Adverse event	*n*	Treatment
Allergic reaction to fresh frozen plasma	1	Antihistamines and prednisolone
Catheter-associated infection	1	Catheter removal and antibiotic treatment
Hypocalcaemia	1	10% calcium gluconate
Dislocation of peripheral vascular access at end of treatment	1	Removal of vascular access
Nausea	2	No specific treatment needed
Coagulation imbalances	2	No specific treatment needed
Moderate arterial hypotension	2	Crystalloid infusion

*n*: number.

**Table 5:** Clinical response to GCS treatment for relapse B in patients with MS.

MS Type at Relapse B	Marked Improvement (*n*)	Moderate Improvement (*n*)	No Effect (*n*)	Deterioration (*n*)
RR-MS (*n* = 29)	9	17	3	0
SP-MS (*n* = 7)	1	6	0	0
PP-MS (*n* = 1)	0	1	0	0
All patients (*n* = 37)	10	24	3	0

*n*: number; deterioration was defined as worsened target neurologic deficit or new neurologic symptoms; marked improvement as clinically significant improvement in function; moderate improvement as a definite change of the neurologic deficit without significant impact on function within the functional score; and no effect as unchanged symptoms.

## Abbreviations

CIS	Clinically isolated syndrome
CNS	Central nervous system
CVA	Central venous access
DMD	Disease modifying drugs
EDSS	Expanded disability status scale
GCS	Glucocorticosteroid
MP	Methylprednisolone
MS	Multiple sclerosis
PP-MS	Primary-progressive multiple sclerosis
RR-MS	Relapsing-remitting multiple sclerosis
SP-MS	Secondary-progressive multiple sclerosis
TND	Target neurologic deficit
TPE	Therapeutic plasma exchange
